# A novel online vaping intervention and smoking prevention program for young adults who vape: protocol for a randomized controlled trial

**DOI:** 10.1186/s13722-025-00566-x

**Published:** 2025-04-21

**Authors:** Denise D. Tran, Jordan P. Davis, Keegan Buch, Adam M. Leventhal, Sarah W. Feldstein Ewing, Eric R. Pedersen

**Affiliations:** 1 Keck School of Medicine, Department of Psychiatry and Behavioral Sciences, University of Southern California, 2250 Alcazar Street, Suite 2200, Los Angeles, CA 90033 USA; 2 RAND Corporation, Santa Monica, CA USA; 3 Keck School of Medicine, Department of Population and Public Health Sciences, University of Southern California, Los Angeles, CA USA; 4 School of Medicine, Departments of Psychiatry and Child Psychiatry. Storrs, University of Connecticut, Connecticut, USA

**Keywords:** E-cigarettes, Smoking, Tobacco, Young adults, Prevention, Cessation, Nicotine, Randomized controlled trial

## Abstract

**Background:**

E-cigarette use (i.e., vaping) is prevalent among young adults in the U.S. Studies show that young adults who vape are more likely to initiate cigarette smoking than young adults who do not vape. Despite this, little research on vaping interventions and prevention of smoking for young adults who vape exist.

**Methods:**

A 2-arm pilot randomized controlled trial (RCT) will be conducted by recruiting young adults ages 18–24 who reported vaping at least once per week in the past 30 days and having never smoked cigarettes at baseline. Participants will be recruited via social media ads and be randomly assigned to an intervention arm, which will be the Live Free From E-cigarettes (LIFFE) mobile-based program (*n* = 50), or a waitlist control arm (*n* = 50). The primary outcomes are biochemically verified 7-day point prevalence abstinence for nicotine vaping, vaping reduction, and smoking susceptibility. Outcomes are measured at 2-, 4-, and 8-weeks after randomization.

**Discussion:**

This is the first RCT to evaluate the effectiveness of a mobile-based intervention that targets smoking susceptibility while also supporting vaping cessation or vaping reduction in young adults. Findings may inform future efforts to prevent transition to cigarette smoking and vaping cessation and reduction in young adults.

**Trial registration:**

ClinicalTrials.gov: NCT06129123; Date of registration: 11/10/2023.

## Background

E-cigarette use, colloquially known as “vaping,” presents a public health concern among young adults (ages 18–24), with the most recent numbers indicating that 10.3% and 15.5% reported current use among those ages 18–20 and 21–24, respectively [1]. These high rates of vaping among young adults are particularly concerning given that long-term health effects of vaping (especially as compared to the effects of combustible cigarettes) are currently not largely understood; however, emerging research and case studies have pointed to potential risks for respiratory, cardiovascular, pulmonary, and oral health issues [2–6]. Young individuals who vape are also at risk for the long-term negative effects that nicotine can have on cognition and neural development, including permanent epigenetic changes in the genome that can be passed to subsequent generations [7]. Nevertheless, young adult e-cigarette users perceive vaping to be of low risk, which may promote continued use [8, 9]. Importantly, vaping also serves as a gateway into combustible cigarette smoking [10–12], with a systematic review indicating consistent direct associations between vaping at baseline and smoking initiation and smoking progression during follow-up [13].

Despite high rates of e-cigarette use in this age group, several studies found that over half of young adult e-cigarette users want to quit vaping [14, 15]. A handful of brief and mobile-accessible interventions have thus been developed to prevent vaping and encourage abstinence from e-cigarettes in young adults [16–20]. For example, a program that delivered text messages over the course of nine weeks was shown to be effective for promoting vaping cessation among young adults [20]. Trials of other programs, such as an app-based intervention [21] and an Instagram-based program are underway [17]. However, additional evidence-based interventions—especially those developed with feedback and qualitative data from young adults—are needed, especially those which address the transition from vaping to smoking combustible cigarettes. At present, there are no known evidence-based treatments that target this phenomenon.

Qualitative research conducted with young adults who vape can provide key information on how to effectively target e-cigarette use. Young adults have responded well to mobile interventions, showcasing promise for this mode of delivery [18–20, 22]. At present, there is limited research examining young adults’ beliefs about what should be included in mobile interventions that target e-cigarette use. However, one study among adolescents who vape found interest in education regarding the health effects of vaping, anecdotes from others who vape, and incentives for cessation [23]. Additionally, a qualitative study led by Tran et al. (2023) investigated young adults’ thoughts on intervention tools that can address vaping as well as the transition to combustible cigarettes [24]. Of note, participants endorsed the inclusions of peer support, cessation progress tracking, education about the harms of e-cigarettes, gamification, and incentivization. Young adults also believed that interventions should include education about the harms of combustible cigarette smoking, teach refusal skills for offers to smoke, and incorporate personal anecdotes from former smokers. It is important that interventions, if they are to be effective, include user feedback from young adults who vape and/or smoke cigarettes.

Personalized normative feedback (PNF) has exhibited promise as a tool for promoting alcohol cessation, including among young adults [25, 26]. Using the styles and techniques of motivational interviewing (MI), PNF helps individuals to reassess and change their behavior by addressing misperceptions of norms regarding said behavior. Although there is no research at present investigating PNF’s efficacy in vaping interventions among young adults, it may be particularly relevant given its emphasis on correcting misperceptions of norms and providing education on young adults’ rates and acceptance of e-cigarette use. Emerging research has indicated that young adult e-cigarette use is associated with their perceptions of their peers’ use and approval of use [27, 28], thus showing support for vaping interventions that address perceived norms.

## Objectives

In a pilot randomized controlled trial (RCT), the present study will test a brief, mobile-accessible intervention primarily built upon PNF to target vaping and the transition to smoking combustible cigarettes among young adults. The primary aim is to determine the efficacy of the program on reducing past 7-day vaping frequency and susceptibility to smoking and increasing biochemically verified 7-day point prevalence abstinence in the immediate term (2-weeks post-randomization) and short-term (4- and 8-weeks post-randomization).

## Methods/Design

### Trial design

This study is a 2-arm pilot RCT with 100 young adults who vape recruited through advertisements placed on social media. Participants will be randomized to the active intervention arm (*n* = 50) or to a waitlist-control arm (*n* = 50).

### Inclusion criteria

Participants are considered eligible if they: [1] are between the ages of 18–24; (b) are able to read English; [3] report vaping at least one day per week in the past month; and [4] report no history of cigarette use at screening and baseline. The inclusion criterion of vaping on at least one day per week in the past month was determined based on existing literature on other vaping intervention trials. Sanchez et al. (2023) and Graham et al. (2021) chose to include those who report using e-cigarettes in the past 30 days [20, 21]. Lyu et al. (2022) and Palmer et al. (2022) chose to include those who report vaping on at least one day per week in the past month and on at least 25 days per month, respectively [17, 18]. PATH data suggest potential low frequency of e-cigarette use, with young adults vaping on an average of 10 days in the past month [16]. Thus, we chose the most moderate inclusion criterion of vaping on at least one day per week.

### Exclusion criteria

Young adults who are currently receiving nicotine cessation services (e.g., counseling services, nicotine replacement therapies, quitline services, self-help materials, apps or other online supports for quitting vaping) will be excluded from the study. Those who report having been diagnosed with a serious mental illness (e.g., schizophrenia or schizoaffective disorder, bipolar disorder), having used an illicit drug in the past 30 days (e.g., cocaine, heroin), or screening positive for an alcohol use disorder in the past year will be ineligible, as they likely require a higher level of care.

### Recruitment and enrollment

Participants will be recruited through online advertisements that will be placed on social media platforms known to be frequented by young adults, such as Instagram and Reddit. Advertisements will briefly describe the study opportunity and include a link to the study’s website that will include a more detailed description of the study’s procedures. The link will also lead interested parties to our screening survey to preliminarily determine their eligibility. Those who are determined to be eligible via this screening survey will be contacted via phone by research personnel to confirm responses to ensure they are not falsifying information and review the consent form to confirm their understanding. Individuals who are considered eligible through both the screening survey and contact with a research team member will be sent a consent form to complete electronically.

### Informed consent

Following eligibility screening procedures, prospective participants must provide informed consent to be enrolled into the study. The electronic informed consent form will provide details about the requirements for participation, LIFFE intervention, voluntary nature of the study, potential risks associated with study participation, incentives, randomization process, and contact information for the study lead and university IRB. Participants will be notified that they may change their mind and discontinue their participation in the study at any time without penalty. After electronically signing the informed consent form, the baseline assessment will be immediately launched.

### Study procedure

**Fig. 1:**
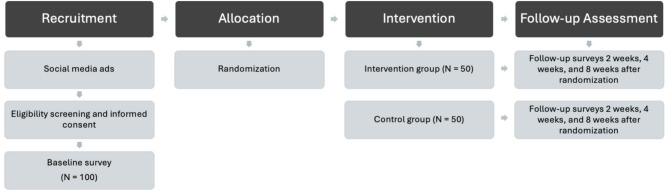
Participant flow of the randomized controlled trial

After signing the consent form, participants will complete a 20-minute baseline survey. Upon completion of the baseline survey, all participants will receive a $25 gift card and be randomized to the intervention (*n* = 50) or the waitlist control condition (*n* = 50). We will utilize stratified randomization to ensure that an equal number of males and females are randomized into each condition. Those randomized into the intervention will be prompted by research personnel to complete the 30-minute intervention, which will be delivered via a webpage accessible through a mobile device. More specifically, participants will be emailed a web link to access the intervention along with personalized log-in information consisting of their email address and a unique 4-digit PIN code. We will collect backend data indicating whether participants have completed all intervention components. We anticipate that most or all participants will complete the intervention given the 100% completion rate in one sitting among those who beta tested our program (completed June 2024; *N* = 20). However, if there are participants who have not completed the full intervention within 24 h of being given access to the program, research personnel will send a text and email reminder. All participants will then complete brief surveys, which will include the same measures from the baseline survey, 2, 4-, and 8-weeks post-randomization (see Fig. 1), to determine the efficacy of the program on reducing vaping and susceptibility to smoking in the immediate term (2-weeks post-randomization) and short term (4- and 8-weeks post-randomization). At most, three text messages and three emails will be sent to remind participants to complete the assessments. Those who do not respond despite the reminder attempts are counted as dropout at each time point. However, participants who do not respond in the previous assessment waves will still be contacted for later assessments. For instance, a participant who did not complete the 2-week follow-up assessment will still be asked to complete the 4-week assessment. Participants will receive a $25 Amazon gift card for completing each survey (up to $100 total). Participants will also be offered raffle tickets for a chance to win various prizes (e.g., movie tickets, $50 Amazon gift card). More specifically, participants will receive one raffle ticket for each survey they complete within two days of receiving the survey via email, giving them the opportunity to earn up to four raffle tickets. Participants who complete all four surveys within two days of receiving them will earn an additional three raffle tickets.

### Development of the intervention

The intervention is called LIFFE (Live Free From E-Cigarettes), which is a 30-minute mobile-accessible interactive program that is personalized to the participant’s individual vaping behavior and beliefs (based on the baseline surveys). Using an iterative process, LIFFE was developed based on evidence supporting PNF and MI in addressing substance use behaviors [25, 29, 30] and recommended best practices for addressing tobacco use and dependence [31]. The first version of LIFFE was informed by our previous qualitative work through a series of focus groups with young adults who vaped, formerly vaped, or vaped and transitioned to smoking [24]. Where possible, we included intervention components that were identified as preferred by young adults who vape, such as teaching participants alternative ways to manage stress, to solicit peer support during quit attempts, and to track their progress with quitting.

The intervention also contains PNF, MI, and education about vaping and smoking harms. The program begins with the MI-based portion, which includes exploration of their experienced health and personal consequences of vaping and potential reasons for and motivation for shifting vaping behaviors (e.g., to save money, to be free from addiction). Drawing on relational methods of MI, the intervention is presented in ways that assume that users know the options that are best for themselves (i.e., “The decision is yours!”) with content that is written in empathetic and encouraging language. We intend to evoke relatedness by building an alliance with the user by also providing opportunities for exploration of options and self-reflection. The intervention will help users to identify reasons, abilities, and opportunities for behavior change (“What are some negatives you have personally experienced since you started vaping?”) and produce change talk (“On a scale from 1–10, with 10 being the highest, how ready [or important or confident] are you to reduce or quit vaping?”). These MI-based content will be used to help move individuals from not considering changing their vaping to considering taking action toward behavior change.

Second, the education portion involves presentation of the actual harms posed by e-cigarettes to clarify any misperceptions of the negative effects of vaping and nicotine exposure in young adults. To address our goal of decreasing susceptibility to smoking, education about the harms of smoking were also included. Based on focus groups with young adults with vaping histories, the education portion included the long-term harms of smoking but especially highlighted the more immediate harms of smoking, which participants reported as more relevant to their age group [24].

Next, users are presented with the PNF portion. In the PNF portion, participants receive feedback about their perceptions of peer vaping by presenting their typical behavior/attitude alongside their perceptions of others’ behavior/attitude alongside the actual norm of the behavior/attitude obtained from the Happiness and Health Study (H&H Study), which comprises of longitudinal cohort data from over 2200 young adults. For example, a participant may see descriptive norms feedback such as “You thought that 40% of people vaped in the past 30 days.” They will then be presented with a figure showing that based on recent research of over 2200 young people, 76.8% of young adult men and 83.6% of young adult women did not vape at all. They will also see that only 4.6% of men and 4.4% of women approve of vaping. Smoking-related normative data are also presented, such as only 2.5% of men and 1.3% of women approve of smoking cigarettes. A brief description of the normative data source (H&H sample) will be provided, which will include the diversity of the sample and help increase believability of the data.

Lastly, strategies recommended for nicotine cessation are presented. For example, in addition the strategies listed previously (e.g., teaching participants alternative ways to manage stress, to solicit peer support during quit attempts, and to track their progress with quitting), participants are taught to select a quit date, identify triggers of their vaping, create incentives for their progress with quitting, and other ways to prepare to quit (e.g., dispose of all vaping-related items prior to quit date). Video testimonials from young adults who formerly vaped, are in the process of quitting, are considering quitting, or vaped and transitioned to smoking are included with tips based on their personal lived experiences with vaping and quitting.

Beta testing of this initial version was recently completed (June 2024) with 20 young adults who reported currently vaping. Using a mixed methods design, both quantitative measures and interviews were used to elicit feedback on the feasibility and acceptability of the program as well as suggestions for improvement. Themes from these interviews have been identified, which will inform the next version of LIFFE for the RCT. Suggested modifications for further improvement, which are being addressed during intervention refinement prior to the RCT, include decreasing the large chunks of texts, having more interactive features (e.g., pop quizzes, ‘check your knowledge’ questions), and having a specific place for users who are motivated to quit to select a quit date (e.g., calendar). Other suggestions that are not feasible to address prior to this pilot RCT will be addressed prior to a subsequent larger RCT, which include having “booster sessions a month or a few months later,” giving users the opportunity to review content and strategies presented during the first session.

### Control group

Participants randomized into the control condition will be instructed to complete the same online surveys (baseline, 2-,4-, and 8-week surveys) sent via email. Control participants will be given the option to access the intervention upon completion of their final assessment at 8-weeks. Along with participants in the experimental group, they will also receive a list of national and local resources for information about the effects of vaping in young people and help with reducing vaping behaviors after completion of the final assessment.

### Measures

#### Screening variables

To screen for eligible participants and to characterize those who enroll into the study, we will gather information about demographics (age, gender, sex at birth, race, ethnicity, college attending status, U.S. region of residence), e-cigarette use (lifetime use, past 30-day use), lifetime cigarette use, interest in quitting e-cigarettes, use of vaping cessation services, and the presence of a serious mental health diagnosis, past month illicit drug use, or past year alcohol use disorder. For the presence of a serious mental health diagnosis and past month illicit drug use, potential participants will respond to a yes/no checklist of serious mental health disorders (e.g., bipolar disorder, schizophrenia) and illicit drugs (e.g., heroin, hallucinogens, cocaine). The Alcohol Use Disorders Identification Test-Consumption (AUDIT-C) will be used to screen for past year active alcohol use disorders with scores greater than four for men and three for women equating to the presence of an alcohol use disorder [32]. The basic demographics will be especially important for exploratory analyses of whether intervention effects differ based on these subgroups.

#### Baseline variables

The baseline survey will include an assessment of our primary and secondary outcomes (described further under Primary and Secondary Outcomes), which include e-cigarette use (e.g., past month use, days of use within the past 7 days), smoking susceptibility, perceptions of vaping- and smoking-related norms, and perceptions of vaping- and smoking-related harms. Further, we will assess for past year vaping quit attempts, motivation to quit vaping, quitting vaping self-efficacy, e-cigarette dependence, and the use of vaping harm reduction strategies. E-cigarette dependence will be measured with the E-cigarette Dependence Scale [33] and the use of vaping harm reduction strategies will be measured with the Vaping Protective Behavioral Strategies Scale [34].

#### Primary outcomes

Our primary outcomes are reductions in past 7-day self-reported vaping frequency (i.e., the number of days they vaped, the number of times per day they picked up their device to vape, and the number of puffs taken before putting their device away per day in the past 7 days), biochemically verified 7-day point prevalence abstinence, and smoking susceptibility using the 4-item Expanded Susceptibility to Smoking Index [35]. At each follow-up assessment, participants who report 7-day abstinence will be mailed a saliva cotinine test kit to provide biochemical verification of their abstinence. Participants will be provided with written instruction on how to perform the saliva cotinine test but will also be contacted by a research team member via a Zoom video call during verification to ensure that participants are able to correctly complete the test. Results will be provided immediately via the test strip and shown to the research team member to verify. Those with a cotinine level of < 30 ng/ml will be reported as having achieved biochemically verified nicotine vaping abstinence. Participants who complete this cotinine test will be compensated with a $20 Amazon gift card.

#### Secondary outcomes

Secondary outcomes for this study are perceived harms of vaping measured using the Short Form Vaping Consequences Questionnaire [36], perceived harms of smoking measured with the Short Form Smoking Consequences Questionnaire [37], motivation to vape (i.e., plan to stop, decrease, continue, or increase vaping), motivation to initiate smoking, and quitting vaping self-efficacy (i.e., 5-point scale assessing confidence about quitting vaping “someday” and “in the next 6 months”). After this study had already been registered to clinicaltrials.gov, we decided to include vaping quit attempts as a secondary outcome based on the current literature [17, 20]. We will measure vaping quit attempts by asking participants whether they have made a quit attempt since their last assessment (yes/no) and to indicate the number of attempts they have made since their last assessment.

#### Covariates

Because the use of other tobacco products and substances are common in young adults [38–41], we will ask participants to report whether they have used other nicotine products (e.g., oral nicotine pouches, cigars, hookah), cannabis, and alcohol at baseline and follow-up timepoints. Those who indicate current use of other nicotine products (aside from combustible cigarettes) will not be excluded, as they may benefit from engaging in vaping cessation efforts. Additionally, because it is possible that participants may have initiated cigarette smoking during their participation in the study, we will also assess for cigarette use during our follow-up assessments.

#### Intervention engagement

To ensure that participants in the intervention arm complete the intervention, we will collect backend data, which will include the percentage of completed content and the number of minutes spent completing the intervention for each participant. These were the same data collected during our initial beta test, with a 100% completion rate among the beta testers in an average of 24.29 min.

### Data analysis plan

#### Primary analysis

Treatment effects of the intervention will be examined using within-subjects repeated measures analysis of variance (RM-ANOVA), which allows for investigations of changes in mean scores over 3 or more time points. Specifically, we will evaluate two models to examine whether there were changes in past 7-day vaping frequency and smoking susceptibility across 3 time points (2-, 4-, and 8-weeks post-randomization) among those who completed the intervention. Logistic regression will be used to examine point prevalence abstinence at our 3 follow-up timepoints. Next, RM-ANOVA will be used to determine whether any changes in past 7-day vaping frequency and smoking susceptibility are the result of the interaction between the condition and time. Past 7-day point prevalence abstinence will be compared between the treatment and control groups using logistic regression. To handle missing data, we will conduct an intent-to-treat (ITT) analysis in which those who were lost to follow-up will be marked as treatment failures.

#### Secondary analysis

Secondary outcomes will also be analyzed using RM-ANOVA. Specifically, treatment effects will be examined by evaluating changes in perceived harms of vaping and smoking, motivation to vape and smoke, quitting vaping self-efficacy, and vaping quit attempts. We will also evaluate whether, compared to controls, participants in the experimental group will report greater perceptions of harms of vaping and smoking, quitting vaping self-efficacy, and vaping quit attempts, and lower levels of motivation to vape and smoke at each follow-up.

#### Exploratory analysis

Analyses will also include an exploration of whether intervention effects differ across subpopulations based on sex (male vs. female), age (< 21 vs. 21 or older), and race/ethnicity. These exploratory moderation analyses may provide information that will allow for further refinement of the intervention as well as to help target the subpopulations for which it is most likely to be effective.

#### Power analysis

Power analyses were performed with power = 0.8 and alpha = 0.05 for small (f = 0.15), moderate (f = 0.25), and large (f = 0.45) effect sizes. With 100 participants, we are adequately powered to detect small (*n* = 62), moderate (*n* = 24), and large (*n* = 10) effects. We anticipate small to medium effects based on previous studies using PNF [42–44].

### Ethics approval

The study protocol was approved by the Institutional Review Board at the University of Southern California (USC IRB). The protocol is registered with ClinicalTrials.gov (protocol # NCT06129123). Any amendments to the protocol will need to be approved by the USC IRB prior to implementing the changes.

### Data monitoring

The Principal Investigator (PI) will be responsible for data safeguarding by implementing the Data and Safety Monitoring Plan. This study will collect data which includes the following information: participant email addresses, phone numbers, and first name. These identifiers are necessary to notify and contact participants about the survey and to send follow-up reminders during the data collection period. PIN codes connecting this identifiable information with confidential data will be randomly assigned to the sample data. The sample data will not be stored in the same database as the survey data, which will be collected by Qualtrics. Thus, survey data will not contain any identifying information. The PIN code is stored together with the answers given by participants. Access to study data will be limited only to the PI and a limited number of study personnel. All individual linkages to data after a participant ends their participation in the study or at the end of the project period will be destroyed.

The PI and the study team will make the determination between adverse events and serious adverse events. All serious adverse events, adverse events, and unanticipated problems will be managed consistent with guidelines set forth by the USC IRB. Any significant adverse events will be reported within 48 h, and all adverse events will be reported on the annual status report in compliance with federal regulations. Participants will be informed that they should contact the PI and/or the USC IRB to report complaints or adverse events.

A Data and Safety Monitoring Board (DSMB) consisting of three members will be created as an independent body with the responsibility of ensuring that the safety of study participants is protected. For example, the DSMB will review any proposed amendments to the study protocol, complete expedited monitoring of all serious adverse events, perform ongoing monitoring of drop-outs and non-serious adverse events, determine whether study procedures should be altered or the study should be paused or terminate for reasons related to the safety of study subjects, and execute periodic review of the completeness and validity of data to be used for analysis of safety and efficacy. The DSMB will also confirm subject privacy and research data confidentiality.

## Discussion

This is the first clinical trial of a mobile-based vaping intervention combined with smoking prevention content for young adults. As vaping among young adults and this risk for future smoking continue to be a concern for tobacco control efforts, this study is timely and urgently needed. LIFFE was developed based on evidence from interventions that addressed other substance use behaviors [25, 30] in young adults, best practices recommended for the treatment of nicotine use and dependence [31], and qualitative work outlining what young adults felt they believed would be essential for vaping cessation or reduction and smoking uptake prevention [24]. Results will be disseminated via publication, scientific meetings, and social media platforms for public viewing.

As technology continues to evolve, it will be important to remain well-informed of ways to utilize available technological platforms to develop innovative strategies to engage a diverse population of young adults in vaping cessation and reduction efforts [45]. A text messaging program was found to be effective for promoting abstinence among young adults who vape [20]. Participants received nine weeks of text messages and had to report interest in quitting vaping in the next 30 days. In mental and behavioral health treatments, longer-term as well as shorter-term treatment options are available, giving patients the opportunity to select treatments that best meet their needs and preferences. Patients who prefer briefer options may find LIFFE to be most feasible. Further, LIFFE was designed to appeal to all young adults who vape, including those who may have never considered quitting or reducing their vaping. This brief model offers an excellent opportunity for these young adults to engage in a “foot-in-the-door” approach or entry to practicing healthy behavior change. Future research should continue to prioritize exploring alternative options or diverse channels to identify the most effective platforms that appeal to diverse youth populations.

### Limitations

It is important to consider potential limitations of the current study. First, this study aims to evaluate the effectiveness of a web-based vaping intervention and smoking prevention program for young adults ages 18–24 and will not include adolescents. Adolescents comprise an age group that is also known to exhibit prevalent use of e-cigarettes and risk for smoking uptake. Efforts are needed to address the needs of at-risk adolescents. Second, we elected to have a waitlist control group given the lack of a comparable vaping intervention for young adults. Having other mobile-based interventions as an active control condition would be ideal. In addition, the use of popular social media platforms for recruitment could potentially limit the generalizability of our young adult sample. Finally, the brevity of the intervention is an innovative feature of this intervention. However, it is not yet clear whether this will limit the longevity of intervention effects. We intend to report whether and when intervention effects decrease or fade over the course of 8 weeks, which can help determine whether and when additional sessions are needed in future iterations of the intervention.

## Conclusions

The public health, scientific, and clinical communities have voiced the importance of curbing vaping rates among young people and increasing resources that support them with cessation. Delivering an intervention that is mobile-accessible, engaging, informative, and brief may be an innovative way to engage young adults in cessation, or at the very least harm reduction, efforts. This pilot study is the first-ever one-session vaping intervention that also aims to reduce susceptibility to smoke in young adults who vape. It aims to rigorously evaluate the effectiveness of a theory-based, empirically informed intervention, with results having potentially strong implications in the development of current and future approaches that address vaping and smoking behaviors among young adults.

## Data Availability

No datasets were generated or analysed during the current study.
